# COVID-19 in a Child With Transposition of the Great Arteries S/P Fontan Palliation: A Case Report and Literature Review

**DOI:** 10.3389/fcvm.2022.937111

**Published:** 2022-07-06

**Authors:** Chen Wen, Guocheng Shi, Wei Liu, Hao Zhang, Gangxi Lin, Huiwen Chen

**Affiliations:** ^1^Department of Cardiothoracic Surgery, Shanghai Children's Medical Center, School of Medicine, Shanghai Jiao Tong University, Shanghai, China; ^2^Department of Pediatrics, The First Affiliated Hospital of Xiamen University, Xiamen, China; ^3^Pediatric Key Laboratory of Xiamen, Xiamen, China; ^4^Institute of Pediatrics, School of Medicine, Xiamen University, Xiamen, China; ^5^The School of Clinical Medicine, Fujian Medical University, Fuzhou, China; ^6^The Third Clinical Medical College, Fujian Medical University, Fuzhou, China

**Keywords:** congenital heart disease, COVID-19, Fontan procedure, SARS-CoV-2, case report

## Abstract

**Background:**

Although adult patients with cardiovascular disease are at higher risk of adverse outcomes such as death or severe infection, limited data exist regarding pediatric patients with congenital heart disease. We would like to report our experience with COVID-19 in a pediatric patient with Fontan circulation. Furthermore, we present a review of patients with Fontan palliation and COVID-19 previously reported in the literature to summarize the clinical characteristics of this population.

**Case Presentation:**

A 9-year-old boy with dextro-transposition of the great arteries, ventricular septal defect, pulmonary stenosis, patent foramen ovale, and borderline left ventricle post bidirectional Glenn shunt and Fontan palliation presented with paroxysmal cough in the context of COVID-19. The coagulation profile was beyond the normal limits, and the patient began to receive anticoagulant aspirin. On the 5th day, the patient presented with fever, sore throat, and fatigue. The oxygen saturations dropped to 93%, and he received nasal catheter oxygen inhalation. On the 7th day, computed tomography of the chest revealed little emerging flaky exudation in the posterior basal segment of the left lower lobe. Nasal cannula was removed on the 12th day, and the coagulation profile returned to normal on the 16th day. After two consecutively negative SARS-CoV-2 viral RNA tests (on the 18th and 19th days, interval ≥ 24 h), he was discharged from the hospital on the 21st day. Literature review indicated that COVID-19 with Fontan palliation seemed to be more common in male adults. Disease presentation varied from mild upper respiratory tract infection to severe pneumonia. Complications were not uncommon in this population. The treatments varied depending on the specific factors. Fortunately, most patients reported a favorable prognosis.

**Conclusion:**

Although patients with COVID-19 and Fontan circulation might have the risk of adverse outcomes due to multiple mechanisms, most patients have a favorable prognosis.

## Introduction

Coronavirus disease 2019 (COVID-19) caused by severe acute respiratory syndrome virus 2 (SARS-CoV-2) has become a global pandemic. Although adult patients with cardiovascular disease are at higher risk of adverse outcomes, such as death or severe infection, limited data exist regarding pediatric patients with congenital heart disease (CHD). Furthermore, children have a lower risk of infection and a milder course than adults (1). Some experts believe that patients with univentricular circulation are susceptible to COVID-19 (2, 3). However, anatomic complexity does not predict infection severity of adult patients with CHD (4). There is a dearth of literature regarding the impact of Fontan circulation on COVID-19, with data mostly based on case reports or series. In this study, we presented the diagnosis and management of COVID-19 in a 9-year-old boy after Fontan palliation. Furthermore, we conducted a review of patients with COVID-19 and Fontan palliation previously reported in the literature to summarize the clinical characteristics of this population.

## Case Description

This boy with a background of dextro-transposition of the great arteries, ventricular septal defects, pulmonary stenosis, patent foramen ovale, and borderline left ventricle presented to our hospital at 3 months of life due to cyanosis. He underwent a bidirectional Glenn procedure at 5 months old. Subsequently, he underwent collateral closure with two embolization coils and enlargement angioplasty of pulmonary arteries with bovine pericardial patch at 4 years old, and completed an extracardiac fenestrated Fontan procedure with a 19 # Gore-Tex conduit at 5^1^/_2_ years old. At the last outpatient visit, his temperature was 37°C; heart rate, 85 beats/min; blood pressure, 110/70 mm Hg; and respiratory rate, 19 breaths/min with oxygen saturation (SpO_2_) 98%. No abnormality was seen in routine blood test, liver and renal function, and the coagulation profile. The echocardiography demonstrated good Fontan circulation, involving a blood flow velocity of 0.58 m/s in the conduit, 0.68 m/s in the inferior vena cava, 0.98 m/s in the cavopulmonary anastomosis, 1.8 m/s at the level of fenestration, and only mild mitral valve regurgitation.

**Figure 1:**
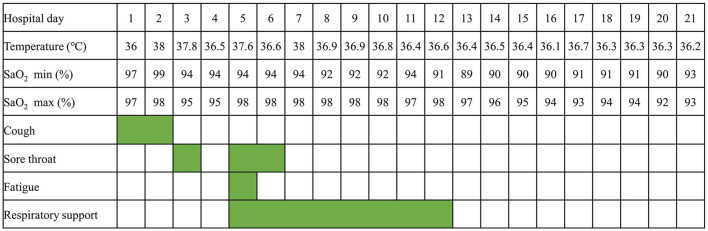
A timeline of the symptoms and managements.

**Figure 2:**
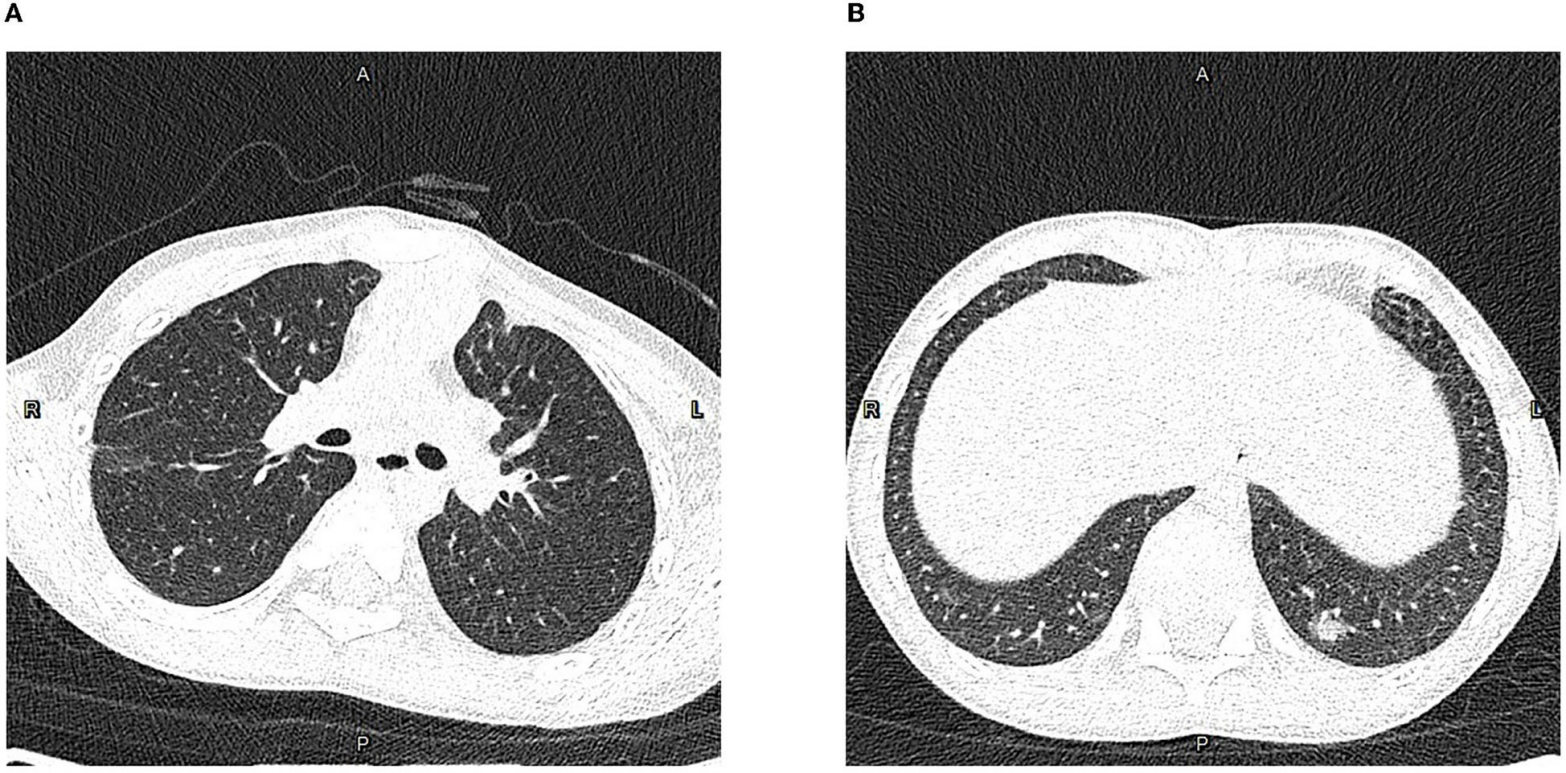
Computed tomography of the chest in the axial plane. **(A)** On hospital Day (HD) 1, showing slight inflammation in the middle lobe of the right lung and upper lobe of the left lung. **(B)** On HD 7, showing little emerging flaky exudation in the posterior basal segment of the left lower lobe.

This 9-year-old patient initially presented with paroxysmal cough (Figure 1). Physical examination revealed pharyngeal congestion and coarse breath sounds in both lungs. Vital signs revealed a temperature of 36°C; heart rate, 102 beats/min; blood pressure, 82/69 mm Hg; and respiratory rate, 20 breaths/min, with SpO_2_ 97%. We informed the patient that the ideal SpO_2_ after Fontan was generally 90–95%. The drop in SpO_2_ caused by COVID-19 would be lower than usual. An electrocardiogram showed a sinus tachycardia with ST-T change and clockwise rotation (Supplementary Figure 1). Computed tomography showed slight inflammation in the middle lobe of the right lung and the upper lobe of the left lung (Figure 2A), which could rule out happy hypoxia. The monocyte count and proportion, and C-reactive protein were elevated. The coagulation profile was beyond the normal limits, including elevated fibrinogen, fibrin degradation products, and D-dimer. Cytokine interleukin (IL)-6 was normal on admission and rose to a peak value on hospital Day 6. Liver and renal functions were normal. Cardiac markers, including creatine kinase and creatine kinase-myocardial band, were within normal limits. The nasopharyngeal swab specimens of the patient, his parents, and older sister were positive for SARS-CoV-2. The strain was the delta variant determined by sequencing. The patient had not been vaccinated against SARS-CoV-2. The prevalent strain was the delta variant at that time.

On HD 2, the patient had fever and complained of sore throat the next day. On HD 5, the patient had a transient low fever, and he presented with sore throat and fatigue. The oxygen saturations dropped to 93%, and he received 1 L/min of oxygen *via* a nasal cannula, which improved the oxygen saturations to 94–96%. Arterial blood gas tests showed decreased CO_2_ partial pressure, O_2_ partial pressure, ${\text{HCO}}_{3}^{-}$, and SpO_2_. The oxygen flow rate was adjusted to 2 L/min, and the oxygen saturations were maintained at 96%. On HD 6, the echocardiography revealed a normal diameter and no filling defect of the inferior vena cava, a blood flow velocity of 0.23 m/s in the portal vein, and only traced mitral valve regurgitation (Supplementary Figure 2). On HD 7, computed tomography of the chest showed little emerging flaky exudation in the posterior basal segment of the left lower lobe (Figure 2B). The patient had a transient low fever at night. On HD 12, nasal cannula was removed. The patient began to receive anticoagulant aspirin on HD 1. Symptoms were observed and related indicators were detected; heparin was not used due to no-significant deterioration. The coagulation profile returned to normal on HD 16. After two consecutively negative SARS-CoV-2 viral RNA tests (on HD 18 and HD 19, interval ≥ 24 h), he was discharged from the hospital on HD 21. We would continue to follow up this patient closely at least for 1 year, including rechecking his clinical manifestations, electrocardiogram, chest computed tomography, and echocardiography.

## Discussion

**Figure 3:**
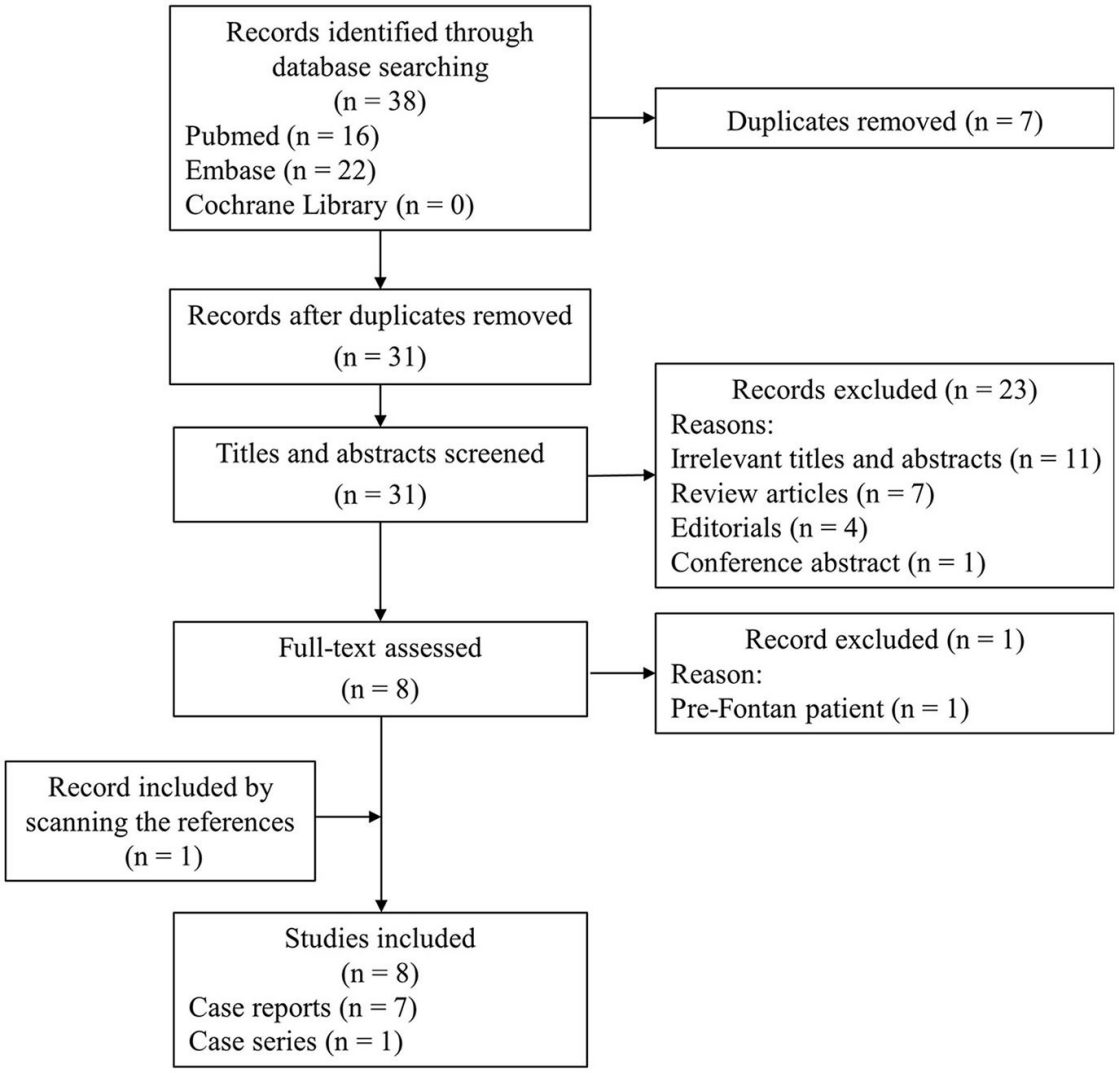
The flow chart of the literature screening process for patients with COVID-19 and Fontan palliation.

Although Fontan palliation with COVID-19 is less common, it has gradually attracted the attention of clinicians. A literature search process using search strategies, comprising a subject word and a free word, was conducted in PubMed, Embase, and Cochrane Library databases (Supplementary Table 1) until April 10, 2022. References of relevant articles were also scanned. The flow chart of the literature screening process is presented in Figure 3. A total of 8 articles involving 14 patients were analyzed. Most pieces of literature are case reports, and Fusco et al. (5) published a case series, describing 7 adult patients. For each case, we extracted the patient's demographics, diagnosis, symptoms, comorbidities, managements, and outcomes (Table 1).

**Table 1:** A summary of articles describing patients with Fontan palliation and COVID-19.

**Study**	**Age**	**Gender**	**Diagnosis**	**Type of Fontan**	**Previous interventions**	**Other comorbidities**	**Signs and symptoms**	**Complications**	**Managements**	**Outcomes**
Vaikunth et al. (6)	40 y	M	TA	Lateral tunnel	Glenn, Fontan	HIV, gout	Fever, shortness of breath, diarrhea	Pneumothorax	Evacuating the pneumothorax, oxygen, nitric oxide, solumedrol, remdesivir, convalescent plasma, draining blood, red cells	Discharged on day 20
Linnane et al. (7)	10 y	M	DILV, PA, ASD, RAA	Extracardiac conduit	Glenn, Fontan	None	Fever, red eyes, lethargy, mild cough	None	Oxygen, cephalosporin, macrolide antibiotics	Discharged on day 14
Bezerra et al. (8)	35 mo	F	HLHS	Extracardiac conduit	Fontan	None	Dyspnea, dry cough	Complete atrioventricular block	Metamizole, heparin, enoxaparin, pleural drain, pacing, pacemaker implantion, milrinone, diuretics, sildenafil, vancomycin, cefepime, oxygen	Discharged on postoperative day 24
Ahluwalia et al. (9)	29 y	M	TA	Lateral tunnel	BT shunt, Glenn, Fontan, device closure of a venovenous collateral, balloon dilation and stent placement of IVC	Fontan Associated Liver Disease under investigation	Low-grade fevers, nonproductive cough, easy fatigability, progressive shortness of breath	None	Oxygen, hydroxychloroquine, azithromycin, furosemide, sildenafil, heparin, lovenox, aspirin	Discharged on day 10
Jicinska et al. (10)	6 y	M	HLHS, dextrocardia	Extracardiac conduit	Fontan	None	Shortness of breath during exercise, increased fatigue during daily activities, newly acquired hepatomegaly	Multiple thrombi	Underwent a thrombectomy, left pulmonary artery plasty, atrial communication enlargement	There were no post-operative thrombotic complications
Jamshidi et al. (11)	51 y	M	TA	Extracardiac Fontan	Atriopulmonary Fontan, extracardiac Fontan	None	Cough, diarrhea	Phlegmasia cerulea dolens	Bilevel positive airway pressure and vasopressor support, remdesivir, tadalafil, warfarin, heparin, convalescent plasma, thrombectomy, left below-the-knee amputation	Discharged to an acute rehabilitation unit
Fusco et al. (5)	1) 24 y 2) 27 y 3) 40 y 4) 39 y 5) 56 y 6) 28 y 7) 23 y	1) M 2) F 3) F 4) M 5) M 6) M 7) F	1) PA IVS 2) AVSD 3) TA 4) DILV with TGA 5) Dextrocardia, TA with TGA 6) TA 7) TA	1) Extracardiac conduit 2) Extracardiac conduit 3) Bjork Fontan 4) Extracardiac conduit 5) Extracardiac conduit 6) Lateral tunnel 7) Extracardiac conduit	1) BT shunt, Glenn, Fontan 2) PA banding, Glenn, Damus-Kaye, Fenestrated Fontan, Fenestration closure 3) Atrial septostomy, BT shunt, Fontan 4) BT shunt, Fontan 5) BT shunt (x2), Glenn, Fontan 6) BT shunt, Glenn, Fontan, stenting of Fontan conduit 7) Glenn, Fontan, stenting of Fontan conduit	1) None 2) None 3) Dysthyroidis, hepatitis C 4) None 5) Restrictive lung disease 6) None 7) PLE, acute kidney injury currently on dialysis, recent hemoperitoneum	1) Malaise, fatigue, sore throat, cough 2) Fever, sore throat, loss of smell, cough 3) Fever, fatigue, myalgia, diarrhoea, cough 4) Fever, fatigue 5) Fever, cough, dyspnoea 6) Fever, cough,myalgia, headache 7) Fever, malaise, fatigue, cough, dyspnoea	1) None 2) None 3) None 4) None 5) None 6) None 7) Desaturation	1) Azithromycin 2) None 3) Azithromycin 4) None 5) Azithromycin 6) Azithromycin 7) Oxygen, steroids, azithromycin	1) Full recovery 2) Full recovery 3) Full recovery 4) Full recovery 5) Full recovery 6) Full recovery 7) Hospitalization required
Chun et al. (12)	51 y	M	TA, PS	-	Fontan	None	Cough, fever, shortness of breath	Phlegmasia cerulea dolens	Vitamin K, convalescent plasma, heparin, enoxaparin sodium, thrombectomy, left below-the-knee amputation, warfarin	Discharged on day 48

*F, female; ASD, atrial septal defect; AVSD, atrioventricular septal defect; DILV, double inlet left ventricle; HLHS, hypoplastic left heart syndrome; IVC, inferior vena cava; M, male; PA, pulmonary atresia; PA IVS, pulmonary atresia with intact ventricular septum; PLE, protein losing enteropathy; PS, pulmonary stenosis; RAA, right aortic arch; TA, tricuspid atresia; TGA, transposition of the great arteries*.

Studies from different countries reporting cases revealed mild-to-severe disease presentation in this population. The virus types were not mentioned in the reviewed cases. In reported cases, COVID-19 with Fontan palliation seemed to be more common in male adults. Different from the demographic data in the literature review, the patient in this report was 9 years old. The symptoms were diverse, with fever and cough being most common. More than half of all the cases were diagnosed with tricuspid atresia. Extracardiac conduit Fontan was mostly common. Our case was diagnosed with transposition of the great arteries and underwent extracardiac fenestrated Fontan. Complications were not uncommon in this population, with thrombotic complications being the most common. No complications were specific to Fontan circulation. No late complications were reported in the reviewed cases. It is worth noting that patients with phlegmasia cerulea dolens were at risk for amputation. Our case had no complications. The treatments varied, depending on the specific factors. More than half of the patients received antibiotics. Fortunately, most patients reported a favorable prognosis. Our patient received anticoagulant and oxygen therapy, and was discharged with full recovery.

There is a need to recognize the potential of SARS-CoV-2 infection in the subpopulation after CHD surgery during the pandemic, particularly when patients present with cough, fever, and hypoxemia that can usually be postoperative morbidities. Since SARS-CoV-2 nucleic acid test was widely carried out in tertiary hospitals, our patient was diagnosed on admission and isolated immediately. It is worth noting that a minority of patients are asymptomatic; the diagnosis is made accidentally before a procedure. As the pandemic continues to evolve, the access to test should be increased, allowing for confirming cases.

When a patient with the Fontan circulation is infected by SARS-CoV-2, the prognosis may be worrying. Owing to the lack of sub-pulmonary ventricular pump, the blood flow through the pulmonary arteries in patients with Fontan physiology is materially driven by the negative intrathoracic pressure and systemic blood pressure. Therefore, patients with Fontan physiology are at increased risk for complications related to coronavirus infection, given the following potential reasons. Any acute respiratory infection may contribute to increased pulmonary vascular resistance, therefore affecting the Fontan circulation. Furthermore, if intubation is required, management for this subgroup of patients is challenging because positive pressure ventilation can cause deleterious effects on the intrapulmonary and intracardiac hemodynamics. Patients with Fontan circulation are likely to have dysfunction of the pulmonary arterial endothelium, which results in impaired NO availability and concomitant increased release of IL-6 (13). Pieces of evidence have indicated that SARS-CoV-2 could determine a more severe cytokines storm in those whose base levels of cytokines are higher and NO levels are lower. Patients with Fontan circulation can have associated liver disease (hepatic fibrosis). The prior study has revealed that patients with liver disease are vulnerable to infection or a severe course of COVID-19 (14). Long-term anticoagulation is usually required in patients with Fontan circulation. Of note, abnormal coagulation is an important aspect of COVID-19, which can result in microthrombus. COVID-19 can lead to myocardial injury, which is a serious complication in patients with univentricular circulation. In addition, myocardial injury and systemic inflammation due to COVID-19 may easily trigger arrhythmia in this susceptible population.

Most patients did not present severe manifestations as expected and were managed routinely. A previous multicenter study also suggested that Fontan palliation did not appear to increase the risk of adverse outcomes (4). Many patients with Fontan palliation were younger, which might explain the favorable results. However, the worse physiological stage, such as cyanosis and pulmonary hypertension, was a predictor for mortality in patients with CHD (4); therefore, Fontan patients with unstable hemodynamics might have adverse outcomes.

## Conclusion

Although patients with COVID-19 and Fontan circulation might have the risk of adverse outcomes due to multiple mechanisms, most patients have a favorable prognosis.

## Data Availability Statement

The original contributions presented in the study are included in the article/Supplementary Material, further inquiries can be directed to the corresponding authors.

## Ethics Statement

The Institutional Review Board of Shanghai Children's Medical Center approved the present case report (approval number SCMCIRB-W2022001; date of approval 7 January 2022). A formal written consent was obtained from the patient's parents for the publication of any potentially identifiable images or data included in this article.

## Author Contributions

CW and GS were responsible for the literature research and the manuscript drafting. GL and HC revised the manuscript for important intellectual content. WL and HZ collected the clinical data. All authors contributed to the article and approved the submitted version.

## Funding

This study is supported by the Chinese National Natural Science Foundation of China (Grant Nos. 82170307, 81801777, and 81970267), Shanghai Jiao Tong University School of Medicine (Grant No. YG2022QN094), and Science and Technology Commission of Shanghai Municipality (Grant Nos. 19411964000 and 20025800300).

## Conflict of Interest

The authors declare that the research was conducted in the absence of any commercial or financial relationships that could be construed as a potential conflict of interest.

## Publisher's Note

All claims expressed in this article are solely those of the authors and do not necessarily represent those of their affiliated organizations, or those of the publisher, the editors and the reviewers. Any product that may be evaluated in this article, or claim that may be made by its manufacturer, is not guaranteed or endorsed by the publisher.

## Supplementary Material

Supplementary Figure 1 12-lead electrocardiogram. Sinus tachycardia, ST-T change.

Supplementary Figure 2 Transthoracic echocardiogram showing **(A)** normal flow in the Fontan circulation and **(B)** mild mitral valve regurgitation.
