# Supplementary material for: COVID-19 in a Child With Transposition of the Great Arteries S/P Fontan Palliation: A Case Report and Literature Review
Source: Front Cardiovasc Med. 2022 Jul 6;9:937111. doi: 10.3389/fcvm.2022.937111 (PMC9297369; doi:10.3389/fcvm.2022.937111)
Supplement: Supplementary file 1 [file Table_1.DOCX]

Supplementary Table 1. Search strategies carried out in PUBMED, EMBASE and Cochrane Library.

PUBMED

1. "COVID-19"[Mesh]

2. COVID-19[Title/Abstract]

3. COVID 19[Title/Abstract]

4. SARS-CoV-2 Infection[Title/Abstract]

5. Infection, SARS-CoV-2[Title/Abstract]

6. SARS CoV 2 Infection[Title/Abstract]

7. SARS-CoV-2 Infections[Title/Abstract]

8. 2019 Novel Coronavirus Disease[Title/Abstract]

9. 2019 Novel Coronavirus Infection[Title/Abstract]

10. 2019-nCoV Disease[Title/Abstract]

11. 2019 nCoV Disease[Title/Abstract]

12. 2019-nCoV Diseases[Title/Abstract]

13. Disease, 2019-nCoV[Title/Abstract]

14. COVID-19 Virus Infection[Title/Abstract]

15. COVID 19 Virus Infection[Title/Abstract]

16. COVID-19 Virus Infections[Title/Abstract]

17. Infection, COVID-19 Virus[Title/Abstract]

18. Virus Infection, COVID-19[Title/Abstract]

19. Coronavirus Disease 2019[Title/Abstract]

20. Disease 2019, Coronavirus[Title/Abstract]

21. Coronavirus Disease-19[Title/Abstract]

22. Coronavirus Disease 19[Title/Abstract]

23. Severe Acute Respiratory Syndrome Coronavirus 2 Infection[Title/Abstract]

24. SARS Coronavirus 2 Infection[Title/Abstract]

25. COVID-19 Virus Disease[Title/Abstract]

26. COVID 19 Virus Disease[Title/Abstract]

27. COVID-19 Virus Diseases[Title/Abstract]

28. Disease, COVID-19 Virus[Title/Abstract]

29. Virus Disease, COVID-19[Title/Abstract]

30. 2019-nCoV Infection[Title/Abstract]

31. 2019 nCoV Infection[Title/Abstract]

32. 2019-nCoV Infections[Title/Abstract]

33. Infection, 2019-nCoV[Title/Abstract]

34. COVID19[Title/Abstract]

35. COVID-19 Pandemic[Title/Abstract]

36. COVID 19 Pandemic[Title/Abstract]

37. Pandemic, COVID-19[Title/Abstract]

38. COVID-19 Pandemics[Title/Abstract]

39. 1 OR 2 OR 3 OR 4 OR 5 OR 6 OR 7 OR 8 OR 9 OR 10 OR 11 OR 12 OR 13 OR 14 OR 15 OR 16 OR 17 OR 18 OR 19 OR 20 OR 21 OR 22 OR 23 OR 24 OR 25 OR 26 OR 27 OR 28 OR 29 OR 30 OR 31 OR 32 OR 33 OR 34 OR 35 OR 36 OR 37 OR 38

40. "SARS-CoV-2"[Mesh]

41. SARS-CoV-2[Title/Abstract]

42. SARS Coronavirus 2[Title/Abstract]

43. Coronavirus 2, SARS[Title/Abstract]

44. Coronavirus Disease 2019 Virus[Title/Abstract]

45. 2019 Novel Coronavirus[Title/Abstract]

46. 2019 Novel Coronaviruses[Title/Abstract]

47. Coronavirus, 2019 Novel[Title/Abstract]

48. Novel Coronavirus, 2019[Title/Abstract]

49. Wuhan Seafood Market Pneumonia Virus[Title/Abstract]

50. SARS-CoV-2 Virus[Title/Abstract]

51. SARS CoV 2 Virus[Title/Abstract]

52. SARS-CoV-2 Viruses[Title/Abstract]

53. Virus, SARS-CoV-2[Title/Abstract]

54. 2019-nCoV[Title/Abstract]

55. COVID-19 Virus[Title/Abstract]

56. COVID 19 Virus[Title/Abstract]

57. COVID-19 Viruses[Title/Abstract]

58. Virus, COVID-19[Title/Abstract]

59. Wuhan Coronavirus[Title/Abstract]

60. Coronavirus, Wuhan[Title/Abstract]

61. COVID19 Virus[Title/Abstract]

62. COVID19 Viruses[Title/Abstract]

63. Virus, COVID19[Title/Abstract]

64. Viruses, COVID19[Title/Abstract]

65. Severe Acute Respiratory Syndrome Coronavirus 2[Title/Abstract]

66. 40 OR 41 OR 42 OR 43 OR 44 OR 45 OR 46 OR 47 OR 48 OR 49 OR 50 OR 51 OR 52 OR 53 OR 54 OR 55 OR 56 OR 57 OR 58 OR 59 OR 60 OR 61 OR 62 OR 63 OR 64 OR 65

67. 39 OR 66

68. "Fontan Procedure"[Mesh]

69. Fontan Procedure[Title/Abstract]

70. Procedure, Fontan[Title/Abstract]

71. Stage 3 Norwood Procedure[Title/Abstract]

72. Fontan Palliation[Title/Abstract]

73. Palliation, Fontan[Title/Abstract]

74. Norwood Procedure, Stage 3[Title/Abstract]

75. Norwood Procedure, Stage III[Title/Abstract]

76. Fontan Operation[Title/Abstract]

77. Operation, Fontan[Title/Abstract]

78. Stage III Norwood Procedure[Title/Abstract]

79. Fontan Circulation[Title/Abstract]

80. Circulation, Fontan[Title/Abstract]

81. Fontan Circuit[Title/Abstract]

82. Circuit, Fontan[Title/Abstract]

83. Hemi-Fontan Procedure[Title/Abstract]

84. Hemi Fontan Procedure[Title/Abstract]

85. Procedure, Hemi-Fontan[Title/Abstract]

86. Norwood Procedure, Stage 2[Title/Abstract]

87. Bidirectional Glenn Shunt[Title/Abstract]

88. Bidirectional Glenn Shunts[Title/Abstract]

89. Glenn Shunt, Bidirectional[Title/Abstract]

90. Stage II Norwood Procedure[Title/Abstract]

91. Bidirectional Glenn Procedure[Title/Abstract]

92. Bidirectional Glenn Procedures[Title/Abstract]

93. Glenn Procedure, Bidirectional[Title/Abstract]

94. Procedure, Bidirectional Glenn[Title/Abstract]

95. Bidirectional Cavopulmonary Shunt[Title/Abstract]

96. Bidirectional Cavopulmonary Shunts[Title/Abstract]

97. Cavopulmonary Shunt, Bidirectional[Title/Abstract]

98. Shunt, Bidirectional Cavopulmonary[Title/Abstract]

99. Norwood Procedure, Stage II[Title/Abstract]

100. Stage 2 Norwood Procedure[Title/Abstract]

101. 68 OR 69 OR 70 OR 71 OR 72 OR 73 OR 74 OR 75 OR 76 OR 77 OR 78 OR 79 OR 80 OR 81 OR 82 OR 83 OR 84 OR 85 OR 86 OR 87 OR 88 OR 89 OR 90 OR 91 OR 92 OR 93 OR 94 OR 95 OR 96 OR 97OR 98 OR 99 OR 100

102. 67 AND 101

EMBASE

1. 'coronavirus disease 2019'/exp

2. 'coronavirus disease 2019':ab,ti

3. '2019 novel coronavirus disease':ab,ti

4. '2019 novel coronavirus epidemic':ab,ti

5. '2019 novel coronavirus infection':ab,ti

6. '2019-nCoV disease':ab,ti

7. '2019-nCoV infection':ab,ti

8. 'coronavirus disease 2':ab,ti

9. 'coronavirus disease 2019':ab,ti

10. 'coronavirus disease 2019 pneumonia':ab,ti

11. 'coronavirus disease-19':ab,ti

12. 'coronavirus infection 2019':ab,ti

13. 'COVID':ab,ti

14. 'COVID 19':ab,ti

15. 'COVID 19 induced pneumonia':ab,ti

16. 'COVID 2019':ab,ti

17. 'COVID-19':ab,ti

18. 'COVID-19 induced pneumonia':ab,ti

19. 'COVID-19 pneumonia':ab,ti

20. 'COVID19':ab,ti

21. 'nCoV 2019 disease':ab,ti

22. 'nCoV 2019 infection':ab,ti

23. 'new coronavirus pneumonia':ab,ti

24. 'novel coronavirus 2019 disease':ab,ti

25. 'novel coronavirus 2019 infection':ab,ti

26. 'novel coronavirus disease 2019':ab,ti

27. 'novel coronavirus infected pneumonia':ab,ti

28. 'novel coronavirus infection 2019':ab,ti

29. 'novel coronavirus pneumonia':ab,ti

30. 'paucisymptomatic coronavirus disease 2019':ab,ti

31. 'SARS coronavirus 2 infection':ab,ti

32. 'SARS coronavirus 2 pneumonia':ab,ti

33. 'SARS-CoV-2 disease':ab,ti

34. 'SARS-CoV-2 infection':ab,ti

35. 'SARS-CoV-2 pneumonia':ab,ti

36. 'SARS-CoV2 disease':ab,ti

37. 'SARS-CoV2 infection':ab,ti

38. 'SARSCoV2 disease':ab,ti

39. 'SARSCoV2 infection':ab,ti

40. 'severe acute respiratory syndrome 2':ab,ti

41. 'severe acute respiratory syndrome 2 pneumonia':ab,ti

42. 'severe acute respiratory syndrome coronavirus 2 infection':ab,ti

43. 'severe acute respiratory syndrome coronavirus 2019 infection':ab,ti

44. 'severe acute respiratory syndrome CoV-2 infection':ab,ti

45. 'Wuhan coronavirus disease':ab,ti

46. 'Wuhan coronavirus infection':ab,ti

47. 1 OR 2 OR 3 OR 4 OR 5 OR 6 OR 7 OR 8 OR 9 OR 10 OR 11 OR 12 OR 13 OR 14 OR 15 OR 16 OR 17 OR 18 OR 19 OR 20 OR 21 OR 22 OR 23 OR 24 OR 25 OR 26 OR 27 OR 28 OR 29 OR 30 OR 31 OR 32 OR 33 OR 34 OR 35 OR 36 OR 37 OR 38 OR 39 OR 40 OR 41 OR 42 OR 43 OR 44 OR 45 OR 46

48 'Severe acute respiratory syndrome coronavirus 2'/exp

49. 'Severe acute respiratory syndrome coronavirus 2':ab,ti

50. '2019 nCOV':ab,ti

51. '2019 new coronavirus':ab,ti

52. '2019 novel coronavirus':ab,ti

53. '2019 severe acute respiratory syndrome coronavirus 2':ab,ti

54. '2019-nCoV':ab,ti

55. 'coronavirus SARS-2':ab,ti

56. 'COVID 19 virus':ab,ti

57. 'HCoV-19':ab,ti

58. 'Human coronavirus 2019':ab,ti

59. 'nCoV-2019':ab,ti

60. 'novel 2019 coronavirus':ab,ti

61. 'novel coronavirus 2019':ab,ti

62. 'novel coronavirus-19':ab,ti

63. 'SARS Coronavirus 2':ab,ti

64. 'SARS-2 (virus)':ab,ti

65. 'SARS-2-CoV':ab,ti

66. 'SARS-CoV-2':ab,ti

67. 'SARS-related coronavirus 2':ab,ti

68. 'SARS2 (virus)':ab,ti

69. 'Sever acute respiratory syndrome coronavirus 2':ab,ti

70. 'Severe acute respiratory coronavirus 2':ab,ti

71. 'Severe acute respiratory syndorme coronavirus 2':ab,ti

72. 'Severe acute respiratory syndrome 2 coronavirus':ab,ti

73. 'severe acute respiratory syndrome 2 virus':ab,ti

74. 'severe acute respiratory syndrome corona virus 2':ab,ti

75. 'severe acute respiratory syndrome coronavirus 2019':ab,ti

76. 'Severe acute respiratory syndrome coronoavirus 2':ab,ti

77. 'Severe acute respiratory syndrome coronvirus 2':ab,ti

78. 'severe acute respiratory syndrome CoV-2 virus':ab,ti

79. 'Severe acute respiratory syndrome related coronavirus 2':ab,ti

80. 'Severe acute respiratory syndrome virus 2':ab,ti

81. 'Severe acute respiratoy syndrome coronavirus 2':ab,ti

82. 'Wuhan coronavirus':ab,ti

83. 'Wuhan seafood market pneumonia virus':ab,ti

84. 48 OR 49 OR 50 OR 51 OR 52 OR 53 OR 54 OR 55 OR 56 OR 57 OR 58 OR 59 OR 60 OR 61 OR 62 OR 63 OR 64 OR 65 OR 66 OR 67 OR 68 OR 69 OR 70 OR 71 OR 72 OR 73 OR 74 OR 75 OR 76 OR 77 OR 78 OR 79 OR 80 OR 81 OR 82 OR 83

85. 47 OR 84

85. 'Fontan procedure'/exp

86. 'Fontan procedure':ab,ti

87. 'Fontan anastomosis':ab,ti

88. 'Fontan conduit':ab,ti

89. 'Fontan connection':ab,ti

90. 'Fontan connections':ab,ti

91. 'Fontan operation':ab,ti

92. 'Fontan operations':ab,ti

93. 'Fontan procedures':ab,ti

94. 85 OR 86 OR 87 OR 88 OR 89 OR 90 OR 91 OR 92 OR 93

95. 85 AND 94

Cochrane Library

1. MeSH descriptor: [COVID-19] explode all trees

2. (COVID-19):ti,ab,kw

3. (COVID 19):ti,ab,kw

4. (SARS-CoV-2 Infection):ti,ab,kw

5. (Infection, SARS-CoV-2):ti,ab,kw

6. (SARS CoV 2 Infection):ti,ab,kw

7. (SARS-CoV-2 Infections):ti,ab,kw

8. (2019 Novel Coronavirus Disease):ti,ab,kw

9. (2019 Novel Coronavirus Infection):ti,ab,kw

10. (2019-nCoV Disease):ti,ab,kw

11. (2019 nCoV Disease):ti,ab,kw

12. (2019-nCoV Diseases):ti,ab,kw

13. (Disease, 2019-nCoV):ti,ab,kw

14. (COVID-19 Virus Infection):ti,ab,kw

15. (COVID 19 Virus Infection):ti,ab,kw

16. (COVID-19 Virus Infections):ti,ab,kw

17. (Infection, COVID-19 Virus):ti,ab,kw

18. (Virus Infection, COVID-19):ti,ab,kw

19. (Coronavirus Disease 2019):ti,ab,kw

20. (Disease 2019, Coronavirus):ti,ab,kw

21. (Coronavirus Disease-19):ti,ab,kw

22. (Coronavirus Disease 19):ti,ab,kw

23. (Severe Acute Respiratory Syndrome Coronavirus 2 Infection):ti,ab,kw

24. (SARS Coronavirus 2 Infection):ti,ab,kw

25. (COVID-19 Virus Disease):ti,ab,kw

26. (COVID 19 Virus Disease):ti,ab,kw

27. (COVID-19 Virus Diseases):ti,ab,kw

28. (Disease, COVID-19 Virus):ti,ab,kw

29. (Virus Disease, COVID-19):ti,ab,kw

30. (2019-nCoV Infection):ti,ab,kw

31. (2019 nCoV Infection):ti,ab,kw

32. (2019-nCoV Infections):ti,ab,kw

33. (Infection, 2019-nCoV):ti,ab,kw

34. (COVID19):ti,ab,kw

35. (COVID-19 Pandemic):ti,ab,kw

36. (COVID 19 Pandemic):ti,ab,kw

37. (Pandemic, COVID-19):ti,ab,kw

38. (COVID-19 Pandemics):ti,ab,kw

39. 1 OR 2 OR 3 OR 4 OR 5 OR 6 OR 7 OR 8 OR 9 OR 10 OR 11 OR 12 OR 13 OR 14 OR 15 OR 16 OR 17 OR 18 OR 19 OR 20 OR 21 OR 22 OR 23 OR 24 OR 25 OR 26 OR 27 OR 28 OR 29 OR 30 OR 31 OR 32 OR 33 OR 34 OR 35 OR 36 OR 37 OR 38

40. MeSH descriptor: [SARS-CoV-2] explode all trees

41. (SARS-CoV-2):ti,ab,kw

42. (SARS Coronavirus 2):ti,ab,kw

43. (Coronavirus 2, SARS):ti,ab,kw

44. (Coronavirus Disease 2019 Virus):ti,ab,kw

45. (2019 Novel Coronavirus):ti,ab,kw

46. (2019 Novel Coronaviruses):ti,ab,kw

47. (Coronavirus, 2019 Novel):ti,ab,kw

48. (Novel Coronavirus, 2019):ti,ab,kw

49. (Wuhan Seafood Market Pneumonia Virus):ti,ab,kw

50. (SARS-CoV-2 Virus):ti,ab,kw

51. (SARS CoV 2 Virus):ti,ab,kw

52. (SARS-CoV-2 Viruses):ti,ab,kw

53. (Virus, SARS-CoV-2):ti,ab,kw

54. (2019-nCoV):ti,ab,kw

55. (COVID-19 Virus):ti,ab,kw

56. (COVID 19 Virus):ti,ab,kw

57. (COVID-19 Viruses):ti,ab,kw

58. (Virus, COVID-19):ti,ab,kw

59. (Wuhan Coronavirus):ti,ab,kw

60. (Coronavirus, Wuhan):ti,ab,kw

61. (COVID19 Virus):ti,ab,kw

62. (COVID19 Viruses):ti,ab,kw

63. (Virus, COVID19):ti,ab,kw

64. (Viruses, COVID19):ti,ab,kw

65. (Severe Acute Respiratory Syndrome Coronavirus 2):ti,ab,kw

66. 40 OR 41 OR 42 OR 43 OR 44 OR 45 OR 46 OR 47 OR 48 OR 49 OR 50 OR 51 OR 52 OR 53 OR 54 OR 55 OR 56 OR 57 OR 58 OR 59 OR 60 OR 61 OR 62 OR 63 OR 64 OR 65

67. 39 OR 66

68. MeSH descriptor: [Fontan Procedure] explode all trees

68. (Fontan Procedure):ti,ab,kw

69. (Fontan Procedure):ti,ab,kw

70. (Procedure, Fontan):ti,ab,kw

71. (Stage 3 Norwood Procedure):ti,ab,kw

72. (Fontan Palliation):ti,ab,kw

73. (Palliation, Fontan):ti,ab,kw

74. (Norwood Procedure, Stage 3):ti,ab,kw

75. (Norwood Procedure, Stage III):ti,ab,kw

76. (Fontan Operation):ti,ab,kw

77. (Operation, Fontan):ti,ab,kw

78. (Stage III Norwood Procedure):ti,ab,kw

79. (Fontan Circulation):ti,ab,kw

80. (Circulation, Fontan):ti,ab,kw

81. (Fontan Circuit):ti,ab,kw

82. (Circuit, Fontan):ti,ab,kw

83. (Hemi-Fontan Procedure):ti,ab,kw

84. (Hemi Fontan Procedure):ti,ab,kw

85. (Procedure, Hemi-Fontan):ti,ab,kw

86. (Norwood Procedure, Stage 2):ti,ab,kw

87. (Bidirectional Glenn Shunt):ti,ab,kw

88. (Bidirectional Glenn Shunts):ti,ab,kw

89. (Glenn Shunt, Bidirectional):ti,ab,kw

90. (Stage II Norwood Procedure):ti,ab,kw

91. (Bidirectional Glenn Procedure):ti,ab,kw

92. (Bidirectional Glenn Procedures):ti,ab,kw

93. (Glenn Procedure, Bidirectional):ti,ab,kw

94. (Procedure, Bidirectional Glenn):ti,ab,kw

95. (Bidirectional Cavopulmonary Shunt):ti,ab,kw

96. (Bidirectional Cavopulmonary Shunts):ti,ab,kw

97. (Cavopulmonary Shunt, Bidirectional):ti,ab,kw

98. (Shunt, Bidirectional Cavopulmonary):ti,ab,kw

99. (Norwood Procedure, Stage II):ti,ab,kw

100. (Stage 2 Norwood Procedure):ti,ab,kw

101. 68 OR 69 OR 70 OR 71 OR 72 OR 73 OR 74 OR 75 OR 76 OR 77 OR 78 OR 79 OR 80 OR 81 OR 82 OR 83 OR 84 OR 85 OR 86 OR 87 OR 88 OR 89 OR 90 OR 91 OR 92 OR 93 OR 94 OR 95 OR 96 OR 97OR 98 OR 99 OR 100

102. 67 AND 101
