# Supplementary figures and images for: COVID-19 in a Child With Transposition of the Great Arteries S/P Fontan Palliation: A Case Report and Literature Review
Source: Front Cardiovasc Med. 2022 Jul 6;9:937111. doi: 10.3389/fcvm.2022.937111 (PMC9297369; doi:10.3389/fcvm.2022.937111)

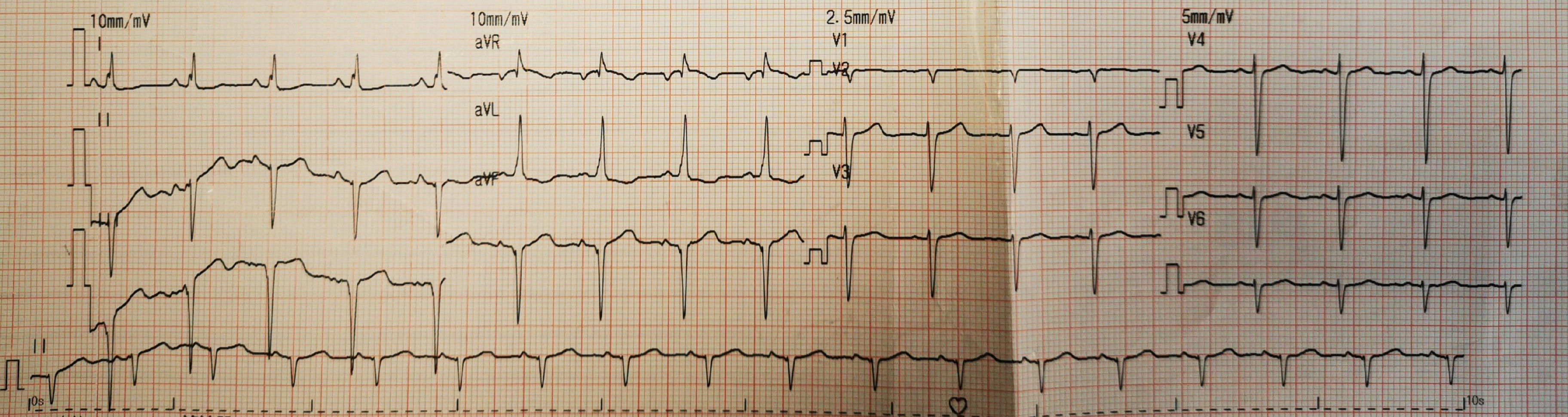

Supplement: Supplementary Figure 1 — 12-lead electrocardiogram. Sinus tachycardia, ST-T change. [file Image_1.tif]
